# Porcelain Aorta in TAVR: Predictor of Adverse Outcomes or Overestimated Risk Factor?

**DOI:** 10.3390/medicina62040699

**Published:** 2026-04-05

**Authors:** Marco Tagliafierro, Darina Kirilina, Ian Mason, Arzhang Fallahi, Julia Baranowska, Jonathan Nickles, Marco Pirelli, Susheel Kodali, Rebecca Hahn, Tamim Nazif, Torsten Vahl, Paul Kurlansky, Michael Argenziano, Arnar Geirsson, Isaac George, Luigi Pirelli

**Affiliations:** Division of Cardiothoracic Surgery, Columbia University Medical Center, 177 Fort Washington Avenue, 7GN-435, New York, NY 10032, USA

**Keywords:** Porcelain aorta, Transcatheter aortic valve replacement, aortic calcification

## Abstract

*Background and Objectives*: Patients with porcelain aorta (PA) pose major surgical challenges during aortic valve replacement, making transcatheter aortic valve replacement (TAVR) the preferred alternative. However, data on the prognostic significance of PA among TAVR recipients are limited. This study sought to evaluate whether PA is associated with adverse short-term outcomes following TAVR. *Materials and Methods*: Consecutive, surgery-naïve patients who underwent TAVR between 2012 and 2020 at a single institution were retrospectively analyzed. Based on preoperative CT scans, patients were categorized as having either porcelain aorta (PA) or non-calcific aorta (NC). Inverse probability of treatment weighting (IPTW) was used to minimize baseline differences, with standardized mean differences (SMD) < 0.1 indicating adequate covariate balance. Logistic regression addressed residual post-IPTW imbalances. *Results*: A total of 2037 patients with severe symptomatic aortic stenosis were identified, of whom 40 (2%) had PA. Compared to the NC population, PA patients were more likely to be younger (*p* = 0.002), had a higher prevalence of heart failure symptoms (*p* = 0.041) and peripheral artery disease (*p* = 0.006). After adjustment for preoperative characteristics, no significant differences were observed between groups in post-TAVR mortality (*p* = 0.498), stroke (*p* = 0.606), or postoperative creatinine levels (*p* = 0.827). However, PA patients experienced significantly longer in-hospital (*p* < 0.001) and ICU (*p* < 0.001) lengths of stay. *Conclusions*: In this single-center cohort, PA did not appear to confer additional risk of mortality, stroke or renal failure, although it remained associated with longer postoperative in-hospital and ICU lengths of stays. TAVR appears to be a safe and effective method of AVR when significant circumferential atherosclerotic aortic calcification precludes aortic cross-clamping.

## 1. Introduction

Porcelain aorta (PA) describes severe, near circumferential or circumferential calcification of the ascending aorta [1]. Diagnosed on preoperative CT scan, it is a high-risk feature for major vascular complications and strokes in patients undergoing open-heart surgery. Several surgical strategies have been designed to mitigate the risk of aortic cannulation and cross-clamping, including the institution of cardiopulmonary bypass (CPB) through non-central alternative arteries (e.g., femoral, axillary), and deep hypothermic circulatory arrest; however, these techniques add complexity and longer pump times, reflecting higher periprocedural morbidity associated with these interventions [2].

In patients with severe aortic stenosis, new alternatives to surgical aortic valve replacement (SAVR) have recently emerged, including transcatheter aortic valve replacement (TAVR). These approaches allow the replacement of the aortic valve without any surgical manipulation of the aorta, thus avoiding cannulation, CPB and cross-clamping. The theoretical advantage of TAVR in PA patients is the lower risk of major aortic complications and embolic strokes. Nevertheless, TAVRs have intrinsic limitations, and implantation of a transcatheter heart valve (THV) in a calcified aorta also carries some risks, including embolization of calcium debris and rupture of the ascending aorta upon valve deployment. There is a paucity of data analyzing outcomes of transcatheter aortic valve interventions in patients with porcelain aorta [3]. Given the heterogeneity of definitions across prior studies, we used a consensus-based visual CT assessment to define porcelain aorta in our cohort, aiming to reflect real-world multidisciplinary heart team practice. Against this background, it remains uncertain whether porcelain aorta itself represents an independent risk factor for adverse early outcomes after TAVR or primarily serves as a marker of global atherosclerotic burden and procedural complexity. We therefore sought to compare short-term outcomes of TAVR in patients with PA versus those with non-calcific aortas (NC) at a high-volume tertiary center, using inverse probability of treatment weighting to account for baseline differences.

## 2. Materials and Methods

### 2.1. Patient Population

Following approval by the institutional ethic committee (Columbia University Institutional Review Board; code: IRB-ACYY0589; latest approval date: 4 February2026; informed consent waived due to the retrospective nature of the study), data were collected retrospectively from the institutional Society of Thoracic Surgeons/American College of Cardiology Transcatheter Valve Therapy (STS/ACC TVT) database, where data are collected prospectively at our institution and are integrated with retrospective chart review as appropriate. All patients undergoing isolated TAVR at Columbia University Medical Center from 2012 to 2020 were included. Based on the extent of near-circumferential or circumferential calcification of the ascending aorta as recorded in our institutional STS/ACC TVT database from preoperative imaging reports (CT scans, transthoracic and transesophageal echocardiography), patients were classified as either porcelain aorta (PA) or non-calcific aorta (NC). Exclusion criteria included patients with history of previous open-heart surgery, patients requiring concomitant interventions, and patients deemed as surgical candidates by multidisciplinary discussion. Preoperative contrast-enhanced CT scans were systematically reviewed as part of the institutional TAVR workup. Imaging interpretation was based on qualitative visual assessment rather than quantitative calcium scoring. CT reports and source images were reviewed by the multidisciplinary Heart Valve team, including cardiac surgeons, interventional cardiologists and cardiac imaging specialists, and porcelain aorta was diagnosed when near-circumferential or circumferential calcification of the ascending aorta was present. In practice, imaging findings were discussed in pre-procedural heart team conferences, and the final classification as PA or NC reflected consensus among team members. No external core laboratory was used; all assessments were performed locally by the heart team.

### 2.2. Endpoints

The primary endpoints were 30-day all-cause mortality and stroke. Secondary endpoints included: in-hospital and ICU lengths of stay, 30-day incidence of aortic valve (AV) reinterventions and postoperative renal function. In PA patients, we also investigated the size of the ascending aorta, the TAVR size and type (balloon- vs. self-expandable), the intraoperative approach and use of embolic stroke protection devices, as well as postoperative myocardial infarction, pacemaker implantation and major vascular complications (as defined by the Valve Academic Research Consortium 3 [VARC 3] [4], i.e., one or more of the following: death, irreversible neurologic impairment, limb or visceral ischemia, or the need for an unplanned surgical or endovascular intervention due to the complication). Procedural characteristics in PA patients, including transfemoral versus alternative (e.g., transapical or other transthoracic, subclavian/axillary, and other non-femoral) access routes, valve type (balloon- vs. self-expandable) and delivery system, anesthesia strategy (general anesthesia vs monitored anesthesia care), and use of cerebral embolic protection devices, were left to operator discretion and were not standardized by protocol; these factors were summarized descriptively within the PA cohort and considered as potential modifiers of procedural complexity, embolic risk, and neurologic and vascular outcomes.

### 2.3. Statistical Analysis

All statistical analyses were conducted using R (version 4.4.2). Normality of continuous variables was assessed using the Shapiro–Wilk test, and all were found to be non-normally distributed (*p* < 0.001). Baseline characteristics were summarized as median and interquartile range (IQR) for continuous variables and as counts with percentages for categorical variables. Group comparisons for continuous variables were performed using the Wilcoxon rank-sum test, while categorical variables were compared using the Chi-squared test or Fisher’s exact test, as appropriate. A *p*-value less than 0.05 was considered statistically significant.

**Figure 1:**
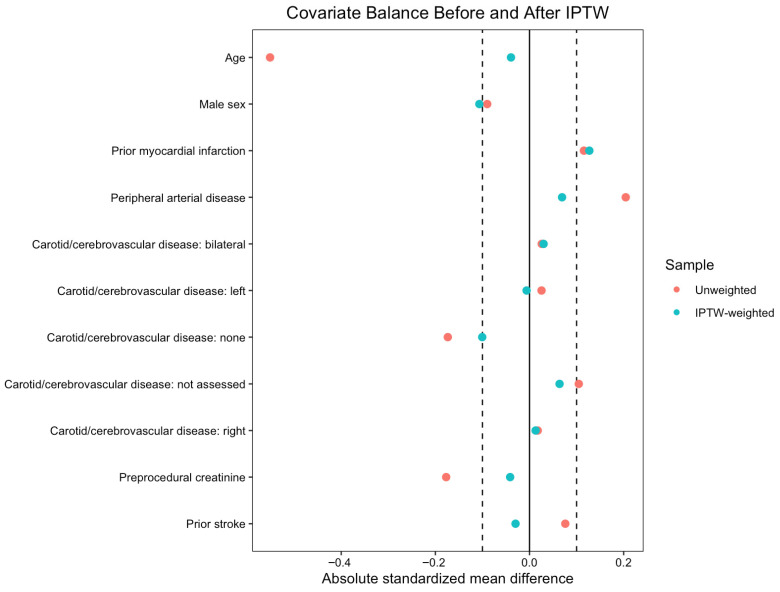
Standardized mean differences for baseline covariates before and after inverse probability of treatment weighting (IPTW) in the overall cohort (**left**) and in patients without prior cardiac surgery (**right**). The vertical reference line at “Mean Differences = 0.1” indicates the prespecified threshold for adequate balance.

Propensity score-based inverse probability of treatment weighting (IPTW) was applied to balance baseline characteristics between patients with and without porcelain aorta. The propensity model included the following covariates: age at procedure, sex, prior myocardial infarction, peripheral vascular disease, severity and laterality of carotid stenosis, pre-procedural creatinine, and history of stroke. Stabilized weights were used to reduce variance. To limit the influence of extreme weights, stabilized IPTW weights were truncated at the 99th percentile. Absolute values of standardized mean difference (SMD) of less than 0.1 were considered indicative of adequate balance (Figure 1). For variables where IPTW did not achieve adequate balance (SMD > 0.1), including prior myocardial infarction, results were further confirmed using multivariable regression models adjusted for the imbalanced covariates. Multivariable IPTW-adjusted regression models were constructed incorporating clinically relevant covariates (prior myocardial infarction, pre-procedural oxygen use, hostile chest phenotype, body mass index, aortic valve peak velocity, mean transvalvular gradient, and aortic insufficiency) to estimate adjusted effect measures for postoperative outcomes. Despite these steps, some covariates, including heart failure symptoms and myocardial infarction history, remained imperfectly balanced after weighting, and all estimates should be interpreted in the context of potential residual confounding factors inherent to retrospective observational data. Logistic regression was used for binary outcomes, and linear regression was applied for continuous outcomes. Results are reported as odds ratios (ORs) for logistic regression models and as mean differences for linear regression models, each with corresponding 95% confidence intervals (CIs) and *p*-values.

## 3. Results

### 3.1. Baseline Characteristics

Based on the inclusion criteria, we identified a total of 2037 patients to be included in the study. Of these, 40 (1.96%) had imaging criteria compatible with the definition of porcelain aorta (PA group). Baseline characteristics can be found in Table 1: patients in the PA group were younger (median [IQR]: 78 [71, 86] vs. 84 [78, 88] years, *p* = 0.002), and had a greater incidence of peripheral artery disease (21 [53%] vs. 617 [31%], *p* = 0.006) and of heart failure (HF) symptoms (53 [93.0%] vs. 2135 [79.8%], *p* = 0.022). Following IPTW (Table 2), only HF symptoms remained significantly different (*p* = 0.007) between the two populations, with all other baseline characteristics being well weighted.

**Figure 2:**
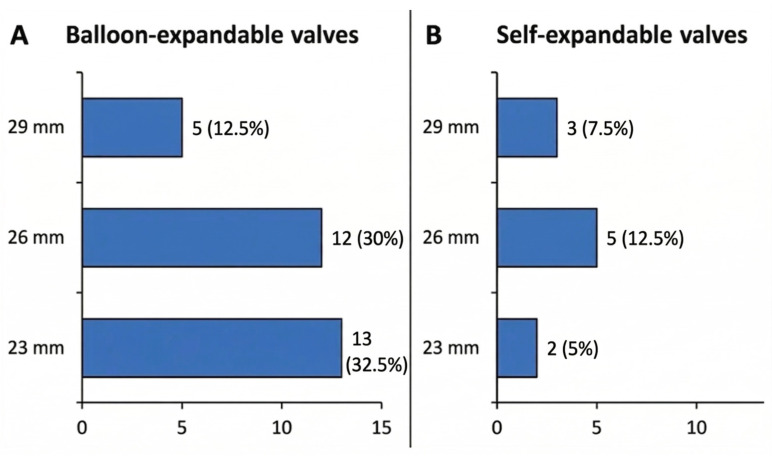
Distribution of transcatheter heart valve sizes in PA patients, stratified by platform: balloon-expandable valves (**A**) and self-expanding valves (**B**). Bars represent the number of PA patients treated with each valve size (mm). Percentages are reported out of the total number of PA patients (i.e., 40).

Thirty (75%) PA patients received a balloon-expandable valve (BEV), with the most common size being 23 mm. Ten (25%) received a self-expandable valve (SEV), with the most common size being 26 mm (Figure 2). Mean (SD) dimension of the ascending aorta was 33 (4.7) millimeters, measured a few cm above the aortic valve annulus at the level of the densest concentric calcium shell (Supplementary Table S1). In PA patients, pre-procedural CT measurements of the aortic annulus and sinotubular junction were routinely obtained, and these dimensions, together with the extent and distribution of calcification, informed the choice between balloon-expandable and self-expanding platforms and the selection of delivery systems. In particular, tapered roots with relatively small sinotubular junctions and dense circumferential calcium favored the use of self-expanding valves to mitigate the risk of aortic injury and excessive interaction between balloons and the calcified aortic wall.

### 3.2. Clinical Outcomes

Intraoperative characteristics and postoperative outcomes following IPTW are shown in Table 3: general anesthesia was employed significantly more often in PA patients than in NC patients (25 [90%] vs. 1175 [59%], *p* = 0.005). Within the PA cohort, transfemoral access was the most common route (27 [76.5%]), with the remainder undergoing alternative non-femoral access (including transapical and other transthoracic approaches), as detailed in Supplementary Table S1. Valve choice, access route, and anesthesia strategy were individualized based on anatomy and operator preference rather than prespecified criteria, and cerebral embolic protection devices were used in only one PA patient (2.5%). No differences were observed between PA and NC groups in 30-day mortality (one [5%] vs. 25 [1%], *p* = 0.498) and stroke (one [5%] vs. 57 [3%], *p* = 0.606).

PA patients had significantly longer median (IQR) ICU (45.3 [26.2, 71.5] vs. 21.0 [0.0, 45.0] hours, *p* < 0.001) and in-hospital (6.0 [3.0, 8.0] vs. 2.0 [2.0, 5.0] days, *p* < 0.001) lengths of stay.

Consistent findings were observed in IPTW-adjusted regression analyses that additionally accounted for covariates that did not achieve adequate balance after weighting (Table 4). No significant differences were found in postoperative mortality (OR: 1.01; 95% CI: 0.97–1.04; *p* = 0.778), stroke (OR: 1.02; 95% CI: 0.93–1.11; *p* = 0.773) and postoperative median (IQR) creatinine (mean difference: 0.04; 95% CI: −0.13–0.21; *p* = 0.827). However, PA patients had a significantly longer ICU stay, with a mean difference of 22.9 h (95% CI: 4.8–40.9; *p* = 0.013), and a longer hospital stay, with a mean difference of 2.1 days (95% CI: 0.8–3.3; *p* = 0.001). Nonetheless, small residual imbalances in clinically relevant characteristics persisted after IPTW, and estimates of association between PA and postoperative outcomes may still be influenced by unmeasured or incompletely adjusted confounders.

Major vascular complications were recorded in four (10%) PA patients: one patient had proximal aortic rupture during valve deployment, while three patients had peripheral vascular complications requiring interventions. Embolic protection devices were employed in one (2.5%) of all PA patients (Supplementary Table S1). One (2.5%) PA patient suffered a myocardial infarction, and two (5%) needed a permanent pacemaker.

## 4. Discussion

As per the 2022 ACC/AHA Aortic Disease Guidelines [5], porcelain aorta is defined as an “extensive, eggshell-like, near-circumferential or circumferential calcification of the intima or media of the aortic wall in the ascending aorta or aortic arch”. The presence of porcelain aorta poses significant challenges in aortic valve replacement due to the heightened risks associated with surgical manipulation, cannulation and cross-clamping of the calcified aorta. Porcelain aorta has been associated with increased morbidity and mortality during conventional SAVR, particularly in relation to perioperative stroke and embolic complications [6]. TAVR has emerged as an attractive alternative, offering a minimally invasive approach that circumvents the need for aortic cross-clamping [3]. In later generation TAVR platforms, the smaller profile and steerable delivery systems allow for less interaction with the aortic wall. Despite this, uncertainty remains over whether PA confers additional procedural risks in TAVR, particularly related to stroke and life-threatening vascular complications. In the PARTNER 1 trial, porcelain aorta, in the absence of additional risk factors, made up 15.1% of their inoperable cohort; however, no subgroup analysis was performed to analyze whether or not this risk factor contributed to a difference in TAVR outcomes [7]. While porcelain aorta is a feature contributing to patient selection for non-surgical approaches, it is unknown whether it is an independent risk factor for worse postoperative outcomes with TAVRs.

The results of this analysis are the following: (1) in this single-center cohort, we did not detect a statistically significant increase in major early clinical adverse events in PA patients compared with NC patients undergoing TAVR, although the small PA sample limits power to exclude moderate differences in risk; (2) patients with PA had significantly longer ICU and in-hospital lengths of stay; and (3) TAVR may represent a safe and effective strategy for aortic valve replacement in patients whose atherosclerotic aortic calcific disease is a contraindication for open-heart surgery, but our findings should be viewed as hypothesis-generating rather than definitive.

Porcelain aorta, defined as significant concentric calcification of the ascending aorta, is a phenotype that describes a peculiar cohort of patients with severe and generalized calcific vascular disease. Not only do these patients show radiologic signs of central calcific pathology involving the aortic valve and the ascending aorta, but they also reveal significant arterial disease in other vascular structures. In the PA cohort, 13% of patients had history of stroke and 53% had history of PAD, highlighting a higher atherosclerotic/calcific burden in this group. Despite these higher risk features, the rate of major vascular complications with TAVR remained low, and not different from the patients without PA, with only 1 patient experiencing aortic complication requiring open repair. Consistently with these findings, rates of myocardial infarction and permanent pacemaker implantation were low in the PA cohort (one and two events, respectively). Post-IPTW analyses did not show any significant differences in aortic valve reinterventions between PA and NC patients (*p* = 0.753), indicating that porcelain aorta did not compromise immediate valve deployment success despite the challenging calcified anatomy. However, given that only 40 patients (2%) were classified as PA, the study may have been underpowered to detect clinically relevant differences in rare hard endpoints such as death or stroke, and absence of statistical significance does not exclude a moderate increase in risk.

The choice of type of THV is also a determinant of risk for aortic injuries. Small, short aortas with concentric calcium may interact with the balloons during deployment of balloon-expandable valves. If the anatomy of the aortic root is “tapered” and the size of the sinotubular junction (STJ) is smaller than the aortic annulus-derived diameter, the balloon can push against a non-compliant rigid structure and cause an injury, resulting in aortic rupture. In our experience, we chose self-expandable valves in the instances in which the balloon size would have been bigger than the calcified STJ, in an effort to minimize the interaction of the balloon with the aortic wall. The low rate of aortic injury during valve deployment might have been the result of a selection of appropriate TAVR technology based on high-risk anatomic features on preoperative imaging. Within the PA cohort, alternative non-transfemoral access and the frequent use of general anesthesia likely reflect the higher procedural complexity associated with extensive aortic calcification and challenging vascular anatomy. These features may partly explain the longer ICU and hospital stays observed in PA patients, even in the absence of a clear increase in early stroke or major vascular complications, while recognizing that we lacked similarly granular procedural data in the NC group to allow direct comparison of access patterns and technical strategies. Similarly, the higher prevalence of heart failure symptoms in PA patients (which persisted as a residual imbalance after IPTW, Table 2) likely contributed to prolonged ICU monitoring and hospital stays, reflecting the greater postoperative care needs in this comorbid subgroup.

Previous studies suggested that balloon-expandable valves might play a role in increasing the risk of strokes in PA cases [8,9]. Nevertheless, we did not encounter any significant difference in the rate of cerebrovascular events based on the type of valve used. However, because valve platform, access route, anesthesia type, and use of cerebral embolic protection were not standardized and cerebral embolic protection (CEP) device use was limited to just few cases, these procedural factors may have modified neurologic risk in ways that our analysis was not powered to fully characterize. Only one patient was treated with a CEP device, as the presence of calcific and atherosclerotic plaques in the subclavian, innominate, and carotid arteries in this vasculopathic cohort could make steering CEP devices and positioning filters challenging and potentially harmful. Studies investigating stroke risk in PA patients undergoing TAVR have presented mixed results. Eckel et al. found no significant difference in stroke rates between PA and NC patients when self-expanding valves were used [8]. Lauten et al. reported higher early stroke rates in PA patients treated with balloon-expandable valves, but the increased risk likely relates more to the technical complexity of valve deployment and manipulation in heavily calcified aortic walls rather than to the specific features of valve design [10].

Moreover, the high calcium burden in the aortic wall increases the risk of embolic complications when using alternative non-transfemoral access. Transapical or subclavian approaches, which may be chosen based on femoral vessels’ anatomical challenges, have been linked to increased stroke risk in porcelain aorta cases [11]. Alternative non-transfemoral accesses, which are typically reserved for patients with more complex anatomy and higher baseline risk, have been associated with worse periprocedural and neurologic outcomes; nevertheless, in our PA cohort the observed stroke rate remained remarkably low. Our results revealed that when the transfemoral approach was used, stroke rates did not differ compared to non-calcified patients, suggesting that porcelain aorta per se does not represent a risk factor for increased aortic wall embolization processes. Taken together, the heterogeneity in procedural strategies in this real-world cohort suggests that our neutral stroke findings should be interpreted within the context of individualized operator decision-making rather than as a uniform procedural approach.

Patients with PA had preoperative creatinine levels within normal limits, and the TAVR procedure did not seem to confer any additional risk for worsening renal function. This can be explained by the fact that calcified aortic structures are often used as radio-opaque reference points during valve implantation, and the necessity of utilizing a larger amount of contrast injection is unusual compared to other non-calcified aortic anatomies. This observation is consistent with data from recent studies, which reveal comparable procedural safety between PA and non-calcified aorta patients [8,12,,13,14].

While major clinical endpoints did not differ, in our study, PA patients required longer ICU and hospital stays, reflecting the greater complexity of postoperative care. More patients with PA required general anesthesia which could have contributed to longer stays. Our observations are consistent with existing evidence that patients with significant calcific aortic disease, regardless of whether they undergo TAVR or SAVR, tend to need longer ICU support and extended hospital stays after valve replacement. Ramirez Del-Val et al. [9] corroborated this trend, showing PA patients spent considerably more time in critical care after SAVR compared to those without calcification. This may be due to the overall vulnerability of the PA population and the added complexity of managing extensive aortic calcification in the perioperative period. These patients are at greater risk for vascular and embolic events, mandating heightened surveillance and a more cautious postoperative approach. In both surgical and transcatheter approaches, current data support that individualized selection of access route, detailed preoperative planning, and careful intraoperative technique are key to minimizing adverse outcomes in this high-risk group. An important consideration is that our analysis focused on early outcomes, including 30-day mortality, stroke, renal function, and length of stay, and did not systematically capture longer-term events or structural valve performance. Thus, our findings primarily inform the acute procedural safety of TAVR in PA rather than its long-term implications, such as late cerebrovascular events, valve durability, or rehospitalizations.

### Limitations

Our study presents several limitations. It is intrinsically limited by its single-center, retrospective design. The small proportion of PA patients not only limits generalizability but also substantially reduces the statistical power to detect differences in infrequent hard endpoints (e.g., mortality, stroke), increasing the risk of type II error and mandating cautious interpretation of neutral findings. Accordingly, the absence of statistically significant differences in early mortality or stroke should not be interpreted as evidence of no effect, but rather as reflecting the limited precision of our estimates in this modest PA cohort. PA status was abstracted from the institutional database without systematic re-review of preoperative images, introducing potential misclassification. While IPTW and logistic regression were used to adjust for baseline differences, residual confounding cannot be entirely excluded; accordingly, associations between PA and clinical outcomes should be interpreted as hypothesis-generating rather than definitive. Our study primarily focused on short-term outcomes; longer-term data on cerebrovascular events, rehospitalizations, and structural valve deterioration were not uniformly available and were therefore not included as formal endpoints. As a result, we cannot draw conclusions regarding the impact of PA on late adverse events after TAVR. Comprehensive echocardiographic assessments were not uniformly available across the cohorts, and therefore not included as formal baseline characteristics or endpoints. Procedural variables such as valve type and access route, anesthesia strategy, and use of cerebral embolic protection were not randomized or standardized and reflect operator discretion, which may introduce selection bias and confound the relationship between PA, neurologic events, and vascular complications. Furthermore, detailed procedural information (including exact access route, delivery system, and sinotubular junction-guided valve selection) was systematically captured only for PA patients, whereas comparable granularity was not uniformly available for NC patients, limiting our ability to perform fully adjusted, procedure-level comparisons between groups. Finally, while we report clinical neurologic outcomes, dedicated neuroimaging data were not available, limiting our ability to detect subclinical embolic events.

## 5. Conclusions

This study suggests that TAVR may be a safe and effective option for patients with porcelain aorta with respect to early outcomes, with no observed increase in major short-term clinical complications compared with patients without extensive calcific disease. Careful patient selection and intraoperative management ensured that adverse events remained low, supporting the procedure’s safety without reliance on these devices. Although PA patients tended to stay longer in the ICU and hospital, suggesting added complexity, their short-term outcomes did not worsen as a result. Moving forward, future research should focus on refining selection criteria and procedural techniques, alongside evaluating long-term valve performance, late cerebrovascular events and rehospitalizations in this vulnerable population. Due to the small sample size of PA patients, the present findings should be considered hypothesis-generating and support the safety of TAVR in carefully selected PA patients, while highlighting the need for larger multicenter studies with longer-term follow-up.

## Figures and Tables

**Table 1:** Baseline clinical and echocardiographic characteristics before inverse probability of treatment weighting (pre-IPTW). All statistically significant *p*-values (<0.05) are highlighted in red. Abbreviations: BMI, body mass index; HF, heart failure; KCCQ, Kansas City Cardiomyopathy Questionnaire; IQR, interquartile range; PA, porcelain aorta; NC, non-calcific aorta.

Baseline Characteristics	NC (*n* = 1997)	PA (*n* = 40)	*p*-Value
Age [years], median (IQR)	84 (78, 88)	78 (71, 86)	0.002
Male, n (%)	949 (48%)	16 (40%)	0.433
BMI [kg/m^2^], median (IQR)	26.3 (23.0, 30.3)	25.8 (22.0, 31.3)	0.562
White ethnicity, n (%)	1869 (94%)	38 (95%)	0.701
Medical history			
Hypertension, n (%)	1851 (93%)	39 (98%)	0.392
Diabetes, n (%)	606 (30%)	16 (40%)	0.255
Chronic lung disease, n (%)			0.878
Mild	225 (11%)	6 (15%)	
Moderate	141 (7%)	3 (8%)	
Severe	132 (7%)	2 (5%)	
None	1499 (75%)	29 (73%)	
Home oxygen, n (%)	131 (7%)	0 (0%)	0.177
Tobacco smoke, n (%)	51 (3%)	2 (5%)	0.645
Cerebrovascular accident, n (%)	189 (10%)	5 (13%)	0.707
Absence of carotid artery disease, n (%)	1055 (53%)	14 (36%)	0.274
Immunosuppression, n (%)	212 (11%)	3 (8%)	0.708
Prior infective endocarditis, n (%)	6 (1%)	0 (0%)	1.00
Peripheral artery disease, n (%)	617 (31%)	21 (53%)	0.006
Myocardial infarction, n (%)	283 (14%)	10 (25%)	0.088
Arrhythmias, n (%)	752 (38%)	18 (45%)	0.433
Heart failure symptoms, n (%)	1551 (78%)	37 (93%)	0.041
KCCQ12, median (IQR)	50.0 (25.5, 72.9)	43.2 (21.8, 57.2)	0.101
Renal status			
Preop creatinine [mg/dL], median (IQR)	1.1 (0.9, 1.4)	1.0 (0.7, 1.3)	0.123
Dialysis, n (%)	81 (4%)	1 (3%)	0.929
STS mortality risk score [%], median (IQR)	4.88 (3.01, 8.00)	5.28 (2.70, 8.00)	0.957
Preop echo data			
Left ventricular ejection fraction [%], median (IQR)	62 (53, 68)	58 (43, 65)	0.048
Primary TAVR indication, n (%)			0.312
Aortic stenosis	1978 (99%)	39 (98%)	
Mixed disease	9 (1%)	1 (3%)	
Aortic insufficiency			
Trivial/Trace	636 (38%)	19 (53%)	
Mild	261 (16%)	6 (17%)	
Moderate	7 (1%)	0 (0%)	
None	764 (46%)	11 (31%)	
Etiology of AV disease, n (%)			0.007
Congenital	2 (0%)	1 (2%)	
Degenerative	1973 (99%)	39 (98%)	
Prior endocarditis	3 (0%)	0 (0%)	
Left ventricular outflow obstruction	4 (0%)	0 (0%)	
Rheumatic fever	9 (0%)	0 (0%)	
Other	6 (0%)	0 (0%)	

**Table 2:** Weighted baseline clinical and echocardiographic characteristics after inverse probability of treatment weighting (post-IPTW). All statistically significant *p*-values (< 0.05) are highlighted in red. Abbreviations: PA, porcelain aorta; NC, non-calcific aorta; BMI, body mass index; HF, heart failure; KCCQ, Kansas City Cardiomyopathy Questionnaire; IQR, interquartile range; STS, Society of Thoracic Surgeons; LVEF, left ventricular ejection fraction; SMD, standardized mean difference.

Baseline Characteristics	NC (*n* = 1981)	PA (*n* = 28)	*p*-Value	SMD
Age [years], median (IQR)	84 (78, 88)	84 (76, 88)	0.843	−0.039
Male, *n* (%)	936 (47%)	10 (37%)	0.261	−0.107
BMI [kg/m^2^], median (IQR)	26.3 (23.0, 30.4)	23.5 (21.4, 29.1)	0.056	−0.198
White ethnicity, *n* (%)	1853 (94%)	27 (97%)	0.770	0.033
Medical history				
Hypertension, *n* (%)	1836 (93%)	27 (99%)	0.054	0.06
Diabetes, *n* (%)	603 (30%)	11 (38%)	0.382	0.077
Chronic lung disease, *n* (%)			0.270	
Mild	225 (11%)	3 (10%)		−0.01
Moderate	140 (7%)	4 (14%)		0.065
Severe	132 (7%)	0 (0%)		−0.057
None	1483 (75%)	21 (75%)		0.002
Home oxygen, *n* (%)	131 (7%)	0 (0%)	0.158	−0.066
Tobacco smoke, *n* (%)	52 (3%)	1 (5%)	0.446	0.026
Cerebrovascular accident, *n* (%)	189 (10%)	4 (13%)	0.602	0.03
Absence of carotid artery disease, *n* (%)	1048 (53%)	12 (43%)	0.732	−0.1
Immunosuppression, *n* (%)	212 (11%)	3 (11%)	0.975	0.002
Prior infective endocarditis, *n* (%)	6 (1%)	0 (0%)	0.771	−0.003
Peripheral artery disease, *n* (%)	619 (31%)	11 (38%)	0.414	0.069
Myocardial infarction, *n* (%)	284 (14%)	8 (27%)	0.063	0.127
Arrhythmias, *n* (%)	747 (38%)	12 (44%)	0.482	0.065
Heart failure symptoms, *n* (%)	1538 (78%)	26 (94%)	0.007	0.165
KCCQ12, median (IQR)	49.4 (25.5, 72.9)	43.8 (21.9, 57.1)	0.102	−0.367
Renal status				
Preop creatinine [mg/dL], median (IQR)	1.1 (0.9, 1.4)	1.1 (0.7, 1.4)	0.505	−0.041
Dialysis, *n* (%)	80 (4%)	1 (5%)	0.867	0.007
STS mortality risk score [%], median (IQR)	4.80 (3.00, 8.00)	8.00 (4.28, 8.89)	0.150	0.194
Preop echo data				
Left ventricular ejection fraction [%], median (IQR)	62 (53, 68)	62 (44, 66)	0.326	−0.138
Primary TAVR indication, *n* (%)			0.954	
Aortic stenosis	1962 (99%)	28 (100%)		0.005
Mixed disease	9 (1%)	0 (0%)		0.00
Aortic insufficiency				
Trivial/Trace	629 (38%)	14 (54%)		0.158
Mild	261 (16%)	5 (19%)		0.035
Moderate	7 (1%)	0 (0%)		−0.004
None	758 (46%)	7 (27%)		−0.189
Etiology of AV disease, *n* (%)			0.976	
Congenital	2 (0%)	0 (0%)		0.003
Degenerative	1957 (99%)	28 (100%)		0.008
Endocarditis	3 (0%)	0 (0%)		−0.002
Left ventricular outflow obstruction	4 (0%)	0 (0%)		−0.002
Rheumatic fever	9 (0%)	0 (0%)		−0.005
Other	6 (0%)	0 (0%)		−0.003

**Table 3:** Intraoperative characteristics and early clinical outcomes after inverse probability of treatment weighting (post-IPTW). All statistically significant *p*-values (<0.05) are highlighted in red. Abbreviations: PA, porcelain aorta; NC, non-calcific aorta; ICU, intensive care unit; LOS, length of stay; AV, aortic valve; IQR, interquartile range.

Intraoperative Variables and Endpoints	NC (*n* = 1981)	PA (*n* = 28)	*p*-Value
Status, urgent, *n* (%)	279 (14%)	6 (20%)	0.272
Anesthesia, *n* (%)			0.005
General	1175 (59%)	25 (90%)	
Moderate sedation	798 (40%)	3 (10%)	
Combination	8 (1%)	0 (0%)	
Primary endpoints			
Mortality, *n* (%)	25 (1%)	1 (5%)	0.498
Stroke, *n* (%)	57 (3%)	1 (5%)	0.606
Secondary endpoints			
In-hospital LOS [days], median (IQR)	2.0 (2.0, 5.0)	6.0 (3.0, 8.0)	<0.001
ICU LOS [hours], median (IQR)	21.0 (0.0, 45.0)	45.3 (26.2, 71.5)	<0.001
AV reoperation, *n* (%)	7 (1%)	0 (0%)	0.753
Postop creatinine [mg/dL], median (IQR)	1.10 (0.86, 1.49)	1.11 (0.80, 1.32)	0.827

**Table 4:** Inverse probability of treatment weighting (IPTW)-adjusted regression analysis of early postoperative outcomes. All statistically significant *p*-values (<0.05) are highlighted in red. Abbreviations: OR, odds ratio; CI, confidence interval; LOS, length of stay; ICU, intensive care unit; PA, porcelain aorta; NC, non-calcific aorta.

Variable	OR/Coeff.	95% CI	*p*-Value
In-hospital mortality, *n* (%)	1.005	0.973, 1.037	0.778
Stroke, *n* (%)	1.016	0.927, 1.114	0.733
In-hospital LOS [days], median (IQR)	2.066	0.805, 3.327	0.001
ICU LOS [hours], median (IQR)	22.869	4.834, 40.903	0.013
Postop creatinine [mg/dL], median (IQR)	0.040	−0.130, 0.211	0.642

Logistic regression was used for binary outcomes (reported as odds ratios with 95% confidence intervals), and linear regression was used for continuous outcomes (reported as mean differences with 95% confidence intervals), all accompanied by corresponding *p*-values.

## Data Availability

The data presented in this study are available on request from the corresponding author.

## Supplementary Materials

The following supporting information can be downloaded at: https://www.mdpi.com/article/10.3390/medicina62040699/s1, Table S1. Intraoperative and early postoperative characteristics of patients with porcelain aorta (PA). All statistically significant *p*-values (<0.05), where applicable, are highlighted in red. Abbreviations: PA, porcelain aorta; TAVR, transcatheter aortic valve replacement; CEP, cerebral embolic protection; IQR, interquartile range; LOS, length of stay.

## Abbreviations

The following abbreviations are used in this manuscript:

Acronym	Meaning
ACC/AHA	American College of Cardiology/American Heart Association
AV	Aortic Valve
AVR	Aortic Valve Replacement
BEV	Balloon-Expandable Valve
CEP	Cerebral Embolic Protection
CI	Confidence Interval
CPB	Cardiopulmonary Bypass
CT	Computed Tomography
HF	Heart Failure
IPTW	Inverse Probability of Treatment Weighting
IQR	Interquartile Range
KCCQ	Kansas City Cardiomyopathy Questionnaire
LOS	Length of Stay
NC	Non-Calcific Aorta
OR	Odds Ratio
PA	Porcelain Aorta
PAD	Peripheral Artery Disease
SAVR	Surgical Aortic Valve Replacement
SD	Standard Deviation
SEV	Self-Expandable Valve
SMD	Standardized Mean Difference
STJ	Sinotubular Junction
STS/ACC TVT	Society of Thoracic Surgeons/American College of Cardiology Transcatheter Valve Therapy
TAVR	Transcatheter Aortic Valve Replacement
THV	Transcatheter Heart Valve
VARC 3	Valve Academic Research Consortium 3
