# Supplementary material for: Porcelain Aorta in TAVR: Predictor of Adverse Outcomes or Overestimated Risk Factor?
Source: Medicina (Kaunas). 2026 Apr 5;62(4):699. doi: 10.3390/medicina62040699 (PMC13117071; doi:10.3390/medicina62040699)
Supplement: Supplementary file 1 [file medicina-62-00699-s001.zip › medicina-4207838-supplementary.pdf]

Supplementary Table

Table S1. Intraoperative and early postoperative characteristics of patients with porcelain aorta (PA). All statistically significant p-values (<0.05), where applicable, are highlighted in red. Abbreviations: PA, porcelain aorta; TAVR, transcatheter aortic valve replacement; CEP, cerebral embolic protection; IQR, interquartile range; LOS, length of stay.

Variable	Value
TAVR deployment approach, n (%)	
Transfemoral	27 (67.5%)
Transthoracic	6 (15%)
Transapical	4 (10%)
Transcarotid	1 (2.5%)
Suprasternal	1 (2.5%)
Transaortic	1 (2.5%)
Embolic protection device used, n (%)	1 (2.5%)
Size of ascending aorta [mm], median (IQR)	33 (4.7)
Major vascular complications, n (%)	4 (10%)
Myocardial infarction, n (%)	1 (2.5%)
Permanent pacemaker, n (%)	2 (5%)
